# A semi-automated and high-throughput approach for the detection of honey bee viruses in bee samples

**DOI:** 10.1371/journal.pone.0297623

**Published:** 2024-03-14

**Authors:** Sofia Levin Nikulin, Poppy J. Hesketh-Best, Dean A. Mckeown, Marla Spivak, Declan C. Schroeder

**Affiliations:** 1 Department of Entomology, University of Minnesota, Saint Paul, Minnesota, United States of America; 2 Department of Veterinary Population Medicine, University of Minnesota, Saint Paul, Minnesota, United States of America; University of Leipzig Faculty of Life Sciences: Universitat Leipzig Fakultat fur Lebenswissenschaften, GERMANY

## Abstract

Deformed wing virus (DWV) was first detected in dead honey bees in 1982 but has been in honey bees for at least 300 years. Due to its high prevalence and virulence, they have been linked with the ongoing decline in honey bee populations worldwide. A rapid, simple, semi-automated, high-throughput, and cost-effective method of screening colonies for viruses would benefit bee research and the beekeeping industry. Here we describe a semi-automated approach that combines an RNA-grade liquid homogenizer followed by magnetic bead capture for total virus nucleic acid extraction. We compare it to the more commonly applied nucleic acid column-based purification method and use qPCR plus Oxford Nanopore Technologies sequencing to evaluate the accuracy of analytical results for both methods. Our results showed high reproducibility and accuracy for both approaches. The semi-automated method described here allows for faster screening of viral loads in units of 96 samples at a time. We developed this method to monitor viral loads in honey bee colonies, but it could be easily applied for any PCR or genomic-based screening assays.

## Introduction

Honey bees are important pollinators of flowering plants and various managed crops [1–3]. Over the past two decades, a large number of reports from different parts of the world show the continued decline in the health of western honey bee, *Apis mellifera*, populations [4–7]. Multiple biotic and abiotic factors such as pests, parasites and pathogens, pesticides, habitat alteration, poor nutrition and lack of genetic diversity were found to contribute to poor colony health [8–10]. Two specific pests and pathogens of honey bees are the parasitic mite, *Varroa destructor*, and the virus, Deformed wing virus (DWV) [11, 12].

The majority of viruses found in the honey bee virome have a single-stranded positive-sense RNA genome (+ssRNA) [13]. In particular, DWV master variants A & B (DWV-A & DWV-B), Acute bee paralysis virus (ABPV), and Israeli acute paralysis virus (IAPV) have been shown to have a major impact on colony survival [14–17]. DWV predominates in many honey bee viromes [13, 18–21]. Pests and pathogens move between colonies within and between apiaries [22–24]. The majority of honey bee viruses cause asymptomatic infections in honey bees [13]; consequently, timely testing for viruses is critical to monitor a colony’s ’ health status [17].

Techniques developed for detecting viruses in honey bees are based on various approaches including enzyme-linked immunosorbent assay (ELISA), oligonucleotide microarray, cell lines, quantitative PCR (qPCR and RT-qPCR) and metagenomic next generation sequencing (mNGS) [25–32]. PCR remains one of the most favored and applied techniques for virus detection and disease diagnosis. It is routinely used for virus diagnostics in many research fields and is a standard for evaluating viral loads in biomedical research, agricultural and environmental sectors [33–38]. NGS technology is an additive and sometimes alternative method, capable of identifying unknown viruses in the sample, including variants and quasispecies [39–41], and is a reliable tool for validation of PCR results [32, 39, 40, 41].

To perform virus screening with qPCR, nucleic acid (RNA or DNA) extraction is often the first step in the screening process. Nucleic acid isolation is initiated with mechanical or chemical disruption of cells, following a purification step by precipitation with ethanol/isopropanol, affinity purification columns or magnetic beads-based technology [33–38, 42, 43]. A common approach in bee research is based on using manual methods of extraction utilizing organic solvents or affinity purification columns kits, or a combination of both. These methods are reliable, but time-consuming [21, 43–48]. Acid phenol RNA extraction based on using organic solvents such as phenol and chloroform, or commercially available TRIzol®, involves using reagents which are highly volatile and toxic [25] and require special and, hence, expensive disposal. In addition, an acid-phenol phase separation method for isolating RNA from DNA requires many steps including multiple steps of incubation, vortexing, centrifugation, rescuing aqueous phase, storing samples at -20C overnight, and washing. As a result, nucleic acid extraction is often a labor intensive and lengthy process [43–45]. Although the affinity column purification method has fewer steps compared to the acid phenol RNA extraction, being based on the manual approach, it is limited in its capacity to provide a rapid solution for large-scale extractions for surveys and diagnostic purposes [21, 46–49]. Moreover, in contrast to the automated approach, manual methods bear a risk of cross-contamination and human-related bias.

The magnetic beads-based technology has been widely used in biomedical research [33–37], and has become one of the most used methods to extract viral RNA for screening for SARS-CoV-2 [36], as rapid diagnosis of COVID-19 is essential to restrict its spreading. A same extraction method combined with an automated approach, which is rapid, high-throughput, uniform and bears low cross contamination risk [50–52] may be employed for honey bee research, in particular for virus screening surveys to screen multiple colonies in a limited timeframe.

The magnetic beads-based technology has previously been used for screening bees for viruses, either for the detection of viruses in individual [53, 54] or pooled samples [53, 55, 56]. However, to our knowledge this method has yet to be compared or validated against the column-based extraction method. Moreover, in all these studies sample preparation remained manual, requiring grinding bees’ samples by hand, and using additional lysis solutions for dissociation of tissues. Here we report on developing a semi-automated high-throughput approach for the detection of honey bee viruses which can be scaled for the simultaneous extraction of 96 pooled bee samples at a time. This method is based on automated dissociation of bee samples in a phosphate buffered saline solution, vacuum manifold-based sterile filtration and a robotic extraction of viral nucleic acids using magnetic beads-based technology. To quantify DWV loads we employ RT-qPCR and confirm our findings by using the Oxford Nanopore Technologies (ONT) GridION sequencing platform.

## Materials and methods

### Samples collection and storage

Samples of 50–100 adult honey bees, *A*. *mellifera* were collected from the colonies located at Agricultural Experimental station in Rosemount, Minnesota, US, during September 2020 and April 2021 (Table 1). Colonies were treated to control *Varroa*: with formic acid pads (Formic Pro®, Mann Lake Ltd) in late August, and oxalic acid dribble in late October. Bee samples were stored at -80°C immediately after washing with 70% Ethanol.

**Table 1:** Forty-eight honey bees pooled samples collected from 20 colonies collected between September 2020 and April 2021.

Apiary	Sample #	Colony #	Date of sampling	extraction method	RNA sample #	DWV-A log10	DWV-B log10
Hill	1	1	23-Sep-20	beads	64	0.0	8.8
	2			columns		0.0	9.4
	3		22-Apr-21	beads	8	0.0	0.0
	4			columns		0.0	0.0
	5	2	23-Sep-20	beads	70	0.0	7.9
	6			columns		0.0	8.8
	7		26-Apr-21	beads	12	0.0	0.0
	8			columns		0.0	0.0
	9	3	23-Sep-20	beads	1	7.5	9.1
	10			columns		8.6	9.5
	11	4		beads	63	6.9	9.1
	12			columns		7.8	9.8
	13		22-Apr-21	beads	22	0.0	0.0
	14			columns		0.0	0.0
	15	5	23-Sep-20	beads	2	9.8	9.7
	16			columns		10.3	9.9
	17	6		beads	78	7.5	7.3
	18			columns		8.4	7.8
	19		22-Apr-21	beads	32	7.3	10.0
	20			columns		8.2	10.6
	21	7		beads	34	8.1	6.4
	22			columns		9.2	7.3
	23	8		beads	50	8.8	0.0
	24			columns		9.6	0.0
	25	9		beads	9	0.0	0.0
	26			columns		0.0	0.0
	27	10	26-Apr-21	beads	29	6.7	0.0
	28			columns		7.6	0.0
	29	11		beads	10	0.0	0.0
	30			columns		0.0	0.0
Kitsune	31	12	29-Apr-21	beads	26	0.0	0.0
	32			columns		0.0	0.0
	33	13		beads	23	0.0	0.0
	34			columns		0.0	0.0
	35	14		beads	24	0.0	0.0
	36			columns		0.0	0.0
	37	15		beads	47	0.0	7.7
	38			columns		0.0	8.3
	39	16		beads	27	0.0	0.0
	40			columns		0.0	0.0
	41	17		beads	37	0.0	0.0
	42			columns		0.0	0.0
	43	18		beads	49	8.3	8.9
	44			columns		9.2	9.2
	45	19		beads	48	0.0	0.0
	46			columns		0.0	0.0
	47	20		beads	25	0.0	8.4
	48			columns		0.0	9.2

### Sample processing

#### Homogenization

Pooled samples of 30 bees representing a single colony (n = 24) were transferred to a 50 ml sterile gentleMACS™ M Tube (Miltenyi Biotec Inc. Auburn, CA, USA), 15 ml of sterile 1X PBS was added and the samples were dissociated using the gentleMACS Dissociator (V1.02, Miltenyi Biotec Inc. Auburn, CA, USA, RNA_02.01). Following centrifugation at 4,700x g, for 5 minutes, at room temperature (RT), 2 ml of the homogenate were transferred to a sterile 2 ml centrifuge tube, and centrifuged at 21,100x g, for 5 minutes, at RT. A 200 μl aliquot of the homogenate was transferred to a sterile 1.5 ml Eppendorf tube for subsequent analysis. All samples were stored at -80°C prior to proceeding to the next step.

#### Filtration

To exclude particles larger than 0.45 um, a filtration step was carried out. 150 μl of previously obtained homogenate from each of the 24 samples were applied to a 0.45 um sterile filtration plate (MultiScreen, MilliporeSigma, USA) and went through a filtration using a Vacuum Manifold (MultiScreen_HTS,_ MilliporeSigma, USA) for a simultaneous filtration of the 24 samples.

#### Viral RNA extraction

To isolate viral RNA, two different approaches were taken: manual isolation with a NucleoSpin Virus kit (Macherey-Nagel, Düren, Germany); and automated extraction using the NucleoMag Virus kit (Macherey-Nagel, Düren, Germany) and a Magnetic Particle Processor (MPP) (KingFisher Flex, Thermo Fisher Scientific, USA). Manufacturer’s instructions were followed for both protocols. For RNA isolation with NucleoSpin kit, 100 ul of sterile 1X PBS was added to 50 ul bee filtrate for a total volume of 150 ul. For NucleoMag kit, 150 ul 1XPBS was added to 50 ul filtrate for a total volume of 200 ul. RNA purity and concentration were assessed with the NanoDrop Spectrophotometer (NanoDrop™, Thermo Fisher Scientific, USA).

#### RT-qPCR

To screen for DWV-A and DWV-B viral loads, Power-Up™ SYBR® Green RNA-to-Ct 1-Step Kit (Applied Biosystems, Foster City, CA, USA) was used. Each reaction was performed in duplicate employing a Bio-Rad real-time PCR machine (CFX96, Bio-Rad, USA) following the ABC assay protocol as described earlier [57]. To quantify viral loads in the RNA samples 5 μl of SYBR mix, 3.92 μl of RNA diluted in molecular grade water to 50 ng/μl, 0.08 μl reverse transcriptase, 0.5 μl (10 pmol) reverse primer (DWV-A or B) and 0.5 μl (10 pmol) universal forward primer were used. Reverse transcription occurred at 45°C for 10 min and denaturation occurred at 95°C for 10 min, followed by 35 cycles of denaturation at 95°C for 15 s, annealing at 58°C for 15 s, and extension at 72°C for 15 s. A high-resolution melt analysis was performed between 72°C and 90°C, at 0.1°C increments, each with a 5 s hold period. Viral genome copies were calculated as described previously [57] and expressed as log10 viral RNA copies per sample.

#### Library preparation and sequencing

To synthesize double stranded cDNA, 10 ul of viral RNA, 1 ul of N6 Primer II A (24 uM, TakaraBio, USA), 1 ul of SMARTer IIA Oligo (24 uM, TakaraBio, USA), 1 ul of 10x Template Switching Reverse Transcriptase (New England Biolabs, MA, USA) for the synthesis of the 1st strand, and 1 ul of Primer IIA (12 uM, TakaraBio, USA) and 1 ul of PrimeSTAR GXL polymerase (TakaraBio, USA) for the synthesis of the 2nd strand were used following manufacturer user manual instructions (TakaraBio, USA; New England Biolabs, MA, USA). The synthesized cDNA was purified using SPRI AMPure beads according to manufacturer’s instructions (TakaraBio, USA), and its integrity and quality were assessed with Qubit 4 Fluorometer (Qubit™4 Fluorometer, Thermo Fisher Scientific, USA) in accordance with One time dsDNA HS assay kit user manual. To prepare the library for nanopore sequencing, the ONT Rapid Barcoding Sequencing Kits (SQK-RPB004, Oxford Nanopore Technologies, UK) were used as per the manufacturer’s guidelines. Libraries were pooled on FLO-MIN106 flow cells and run on the GridION. Sequencing performance was monitored and was terminated after 24 h.

#### Metagenomic analysis

Sequencing reads were filtered to a minimum length (≥200 bp) and Q-value (≥9) by MinKNOW v4.3.4. Basecalling and demultiplexing was performed using Guppy v6.4 with the high accuracy model. Guppy is only available to NanoPore customers through their community site (https://community.nanoporetech.com). PoreChop v0.2.4 [58] was used to remove the nanopore barcode adapter sequences. To assemble metagenome-assembled contigs, the quality filtered reads were assembled by Canu v2.2 [59] using the following assembly parameters: *-nanopore maxInputCoverage = 2000 corOutCoverage = all corMinCoverage = 0 corMhapSensitivity = high minoverlap = 50 minread = 200 genomesize = 5000*. Of 48 viromes, 42 were successfully assembled to generate contigs (24 NucleoSpin® and 18 NucleoMag™). Contigs with a minimum length of 1 kbp were binned manually with the anvi’o v7.1 [60, 61] interactive interface.

Briefly, anvi’o profiled the contigs using Prodigal v2.6.3 [62], with default parameters, then reads were mapped to the contig database using Minimap2 v2.24 [63], and the read recruitment was stored as a BAM file using samtools. Anvi’o profiles each BAM file, estimating the read coverage and detection statistics of each contig, we then normalized coverage as Reads per kilobase of transcript per Million reads mapped (RPKM) using the python package bioinforkit v2.1.0 [64]. Coverage and RPKM was combined into a merged profile database. The contigs database was populated with additional data, incorporating HMMER results against Virus Orthologous Groups (VOGs; https://vogdb.org/) in addition to the standard anvi’o HMMR profiles, NCBI COGs and KEGG KOfam database [65]. Contig taxonomy was predicted by running Kraken2 v2.1.2 [66, 67] using the non-redundant NCBI database on the gene calls. Finally, merged profiles were clustered with the automatic binning algorithm CONCOCT, and the anvi’o profile was visualized for manual binning. Binning was guided by sequence composition similarity (visualized as a dendrogram in the Anvi’o interface), and the presence of viral HMM hits to the VOG database.

#### Validation of honey bee RNA virus genomes from binned contigs

Binned contigs were size filtered to 5 kbp, then aligned to reference genomes using Minimap2. References for complete genomes included DWV-A (NC_004830.2) and DWV-B/VDV-1 (NC_006494.1). Alignments were used to identify probable viral genomes. Prodigal was utilized to identify coding regions and regions were annotated using BLASTn (Nucleotide Basic Local Alignment Search Tool).

#### Data analysis and visualization

Data was visualized and statistically analyzed using *R* v4.1.0 in RStudio build 576 and Microsoft Excel software. Statistical tests were performed using base features in *R* and data visualized with the package *ggplot2* v3.4.1. Numeric values of the read length, number of reads and average quality of each read was acquired using SeqKit v2.3.0 [68]. Welch approximation t-test was used to compare data generation outputs from NucleoSpin® versus NucleoMag™ (i.e., RNA yields, number of reads generated, read length, and average read quality, average read mapped to reference genomes).

## Results

### RNA extraction and nanopore sequence data generation

**Fig 1:**
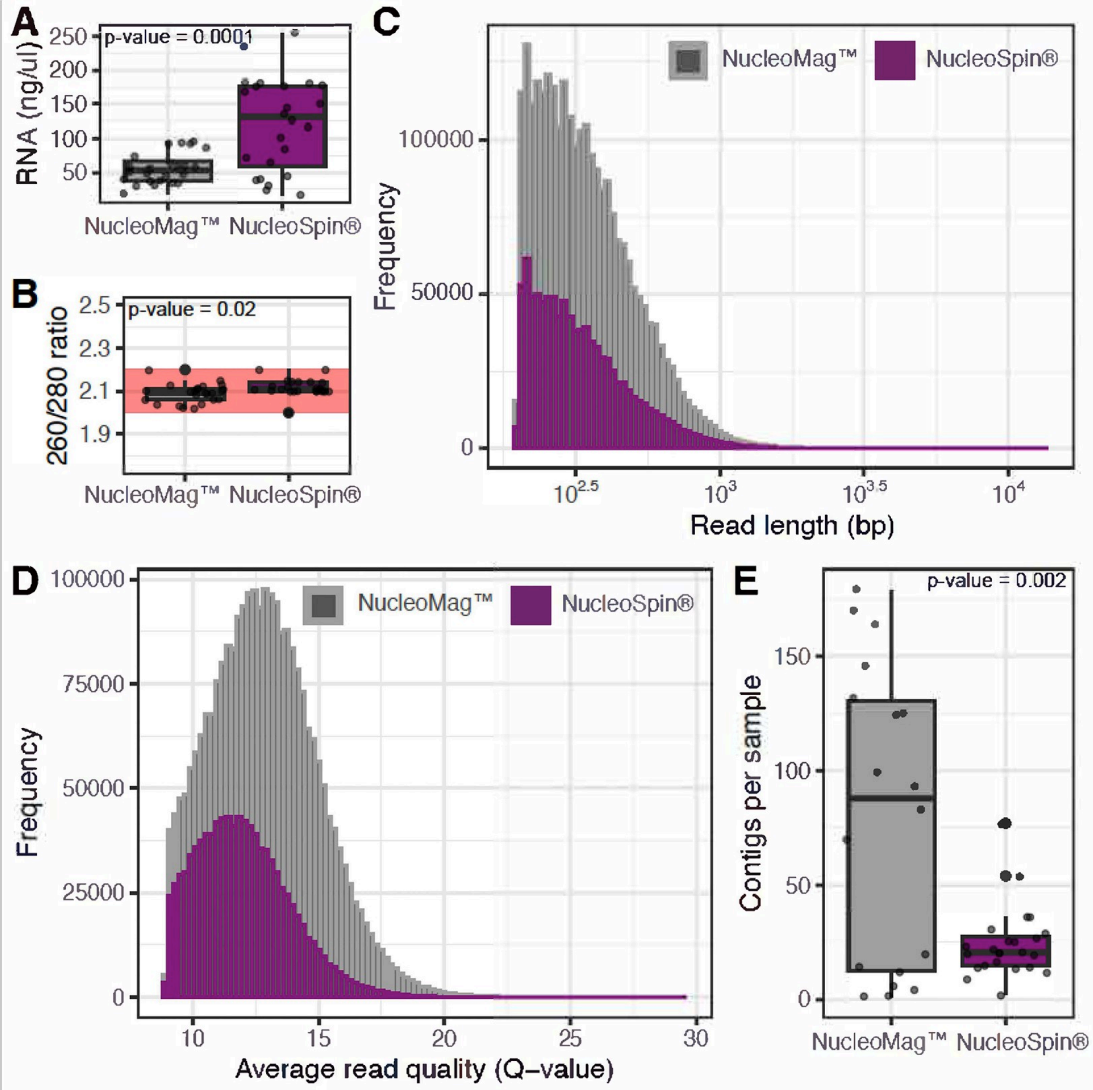
RNA extraction with NucleoMag™ is comparable, and by some measure preferable, to NucleoSpin®. Overview of the RNA quantity and quality as represented by (A) RNA concentration (ng/μl) and (B) quality measured by spectrophotometry (260/280 ratio). Opaque red zone indicates the range of RNA purity (~2–2.2) which is the best practice quality to ensure the greatest likelihood of sequencing success. Histograms with bin size of 100 of (C) read lengths, and (D) average read quality generated from RNA extracted by either NucleoMag™ (dark gray) or NucleoSpin® (magenta), and the (E) number of *de novo* contigs assembled by CANU.

RNA was isolated twice from the set of 24 honey bee pooled samples each representing a colony, once using a manual column affinity method (NucleoSpin®), and second time employing an automated magnetic beads technology-based method (NucleoMag™). In total RNA was extracted from 48 honey bee pools (Table 1), and the yield was quantified (ng/μl) and assessed for quality (260/280 and 260/230 ratios, S1 Table). The NucleoMag™ protocol did not have comparable yields to NucleoSpin® (t(45) = -4.4263, p-value = 0.0001; Fig 1A), or purity represented as 260/280 ratios (t(45) = -2.329, p-value = 0.0259; Fig 1B), as assessed by Welch t-test (S2 Table). Despite the differences between the means, both methods fell within an acceptable range that ensures the greatest likelihood of successful sequencing. Furthermore, NucleoMag™ benefited from a high consistency in RNA yields. Overall, we observed a similar distribution between the two datasets in regard to read length and quality (Fig 1C and 1D), with NucleoMag™ generating a greater abundance of data. Honey bee cDNA samples were sequenced, generating on average 41,098 and 113,719 reads per sample from NucleoSpin® and NucleoMag™, respectively. A total of 2,024 contigs were assembled from the dataset, with more contigs assembled from samples extracted with NucleoMag™ (1,444) than NucleoSpin® (580) (Fig 1E). To explore this dataset further we conducted multiple Welch t-tests, comparing the means of the two extraction protocols for the number, quality and the length of sequenced reads and contigs. Of the five tests conducted, four revealed significant differences between the means of the two groups (*p*-value <0.05) (S2 Table). Specifically, we found differences between the means of NucleoMag™ and NucleoSpin® on: the number of reads generated (t(45) = 3.4161, *p*-value = 0.001); read length (t(100275) = -8.443659, *p*-value = <2.2x10-16); average read quality (t(100998) = 69.5, *p*-value = <2.2x10-16) and number of contigs assembled per sample (t(18.427) = 3.5612, *p*-value = 0.002; Fig 1E). No differences between the means were observed in contig lengths (t(771.86) = -0.01815, *p*-value = 0.9855). With the exception of read length and contig length, we observe the trend of NucleoMag™ having higher means as compared to column extraction method.

#### RT-qPCR

**Fig 2:**
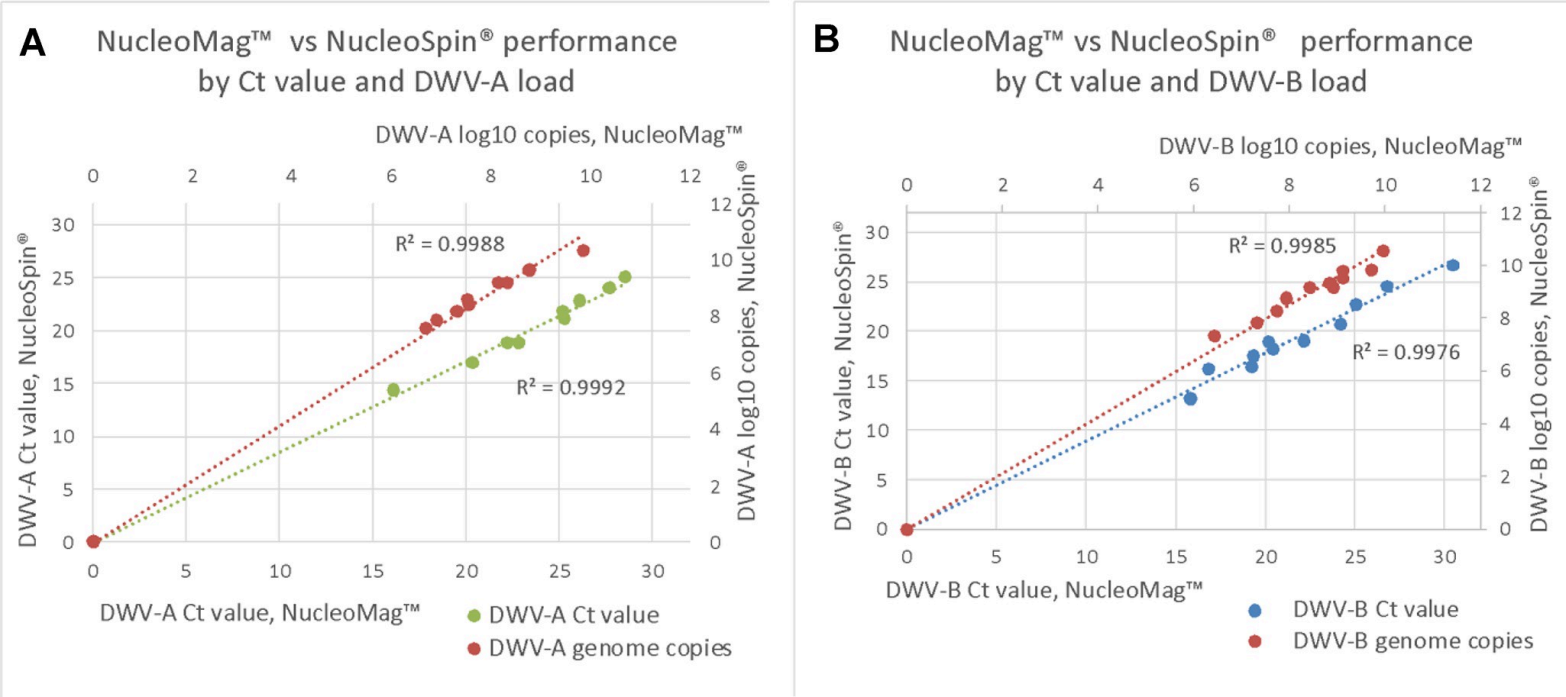
Correlation between the two methods of extraction by *C*_*t*_ value and log10 viral genome copies per colony. (A) R^2^ = 0.9988 for viral genome copies, R^2^ = 0.9992 for *C*_*t*_ values for DWV-A; (B) R^2^ = 0.9985 for viral genome copies, R^2^ = 0.9976 for *C*_*t*_ values for DWV-B.

Forty-eight RNA samples were screened for DWV-A and DWV-B variants’ presence and quantity applying RT-qPCR-based ABC assay [57]. Both methods provided similar analytical results with a slight difference in the strength of amplification signal reflected as 1.0–1.1 in log10 viral genome copies per colony more for RNA isolated with NucleoSpin® compared to NucleoMag™. The analysis of correlation between the *C*_t_ values obtained from both RNA sets, as well as between viral genome copies per colony showed a high correlation for both DWV strains (R^2^ = 0.9992 and R^2^ = 0.9988 for DWV-A; R^2^ = 0.9976 and R^2^ = 0.9985 for DWV-B, for *C*_t_ values and viral genome copies respectively, Fig 2).

Out of 24 the colonies 11 were confirmed to be DWV-A and DWV-B free or below the limits of detection. All 11 colonies were sampled in April 2021 and included colonies 1, 2, 4, 9, 11–14, 16, 17 and 19, corresponding to RNA samples 8, 12, 22, 9, 10, 26, 23, 24, 27, 37 and 48, respectively (Table 1). Three out of these colonies–colony 1, 2 and 4 (RNA samples 64, 70 and 63 respectively, Table 1)—were sampled in fall 2020 as well. Colonies 1 and 2 showed moderate to high levels of DWV-B, (RNA samples ## 64 and 70, Table 1) showing 8.8 (NucleoMag™) and 9.4 (NucleoSpin®), and 7.9 (NucleoMag™) and 8.8 (NucleoSpin®) log10 DWV-B copies for colony 1 and 2, respectively (Table 1). Colony 4 was positive for both DWV-A and DWV-B (6.9 and 9.1 (NucleoMag™), and 7.8 and 9.8 (NucleoSpin®), respectively, corresponding to RNA sample 63, Table 1). Another colony sampled twice, in fall 2020 and spring 2021, colony 6, was found positive for both DWV master variants A (7.5 (NucleoMag™) and 8.4 (NucleoSpin®) in fall 2020; 7.3 (NucleoMag™) and 8.2 (NucleoSpin®) in spring 2021) and B, increasing drastically its DWV-B loads in spring 2021 (7.3 (NucleoMag™) and 7.8 (NucleoSpin®) in fall 2020; 10 (NucleoMag™) and 10.6 (NucleoSpin®) in spring 2021), (Table 1). Two more colonies sampled in September 2020 only, colony 3 and 5, showed moderate to high levels of DWV-A (7.5 (NucleoMag™) and 8.6 (NucleoSpin®), and 9.8 (NucleoMag™) and 10.3 (NucleoSpin®) log10 genome copies for colony 3 and 5, respectively); and high loads of DWV-B (9.1 (NucleoMag™) and 9.5 (NucleoSpin®), and 9.7 (NucleoMag™) and 9.9 (NucleoSpin®) log10 genome copies for colony 3 and 5 respectively), corresponding to RNA samples 1 and 2 (Table 1). In summary, fall virus levels observed were relatively high as expected. The rest of the colonies were sampled in April 2021 only, showing presence of either both DWV master variants A and B (colonies 7 and 18), or A (colonies 8 and 10), or B (colonies 15 and 20). These spring viral quantities observed were relatively high for the season, ranging from 6.7 to 8.8 (NucleoMag™) and 7.6 to 9.6 (NucleoSpin®), and 6.4 to 8.9 (NucleoMag™) and 7.3 to 9.2 (NucleoSpin®), log10 DWV-A and DWV-B genome copies per colony respectively (Table 1, RNA samples 7, 8, 10, 15, 18, 20).

#### Coverage across DWV genomes

**Fig 3:**
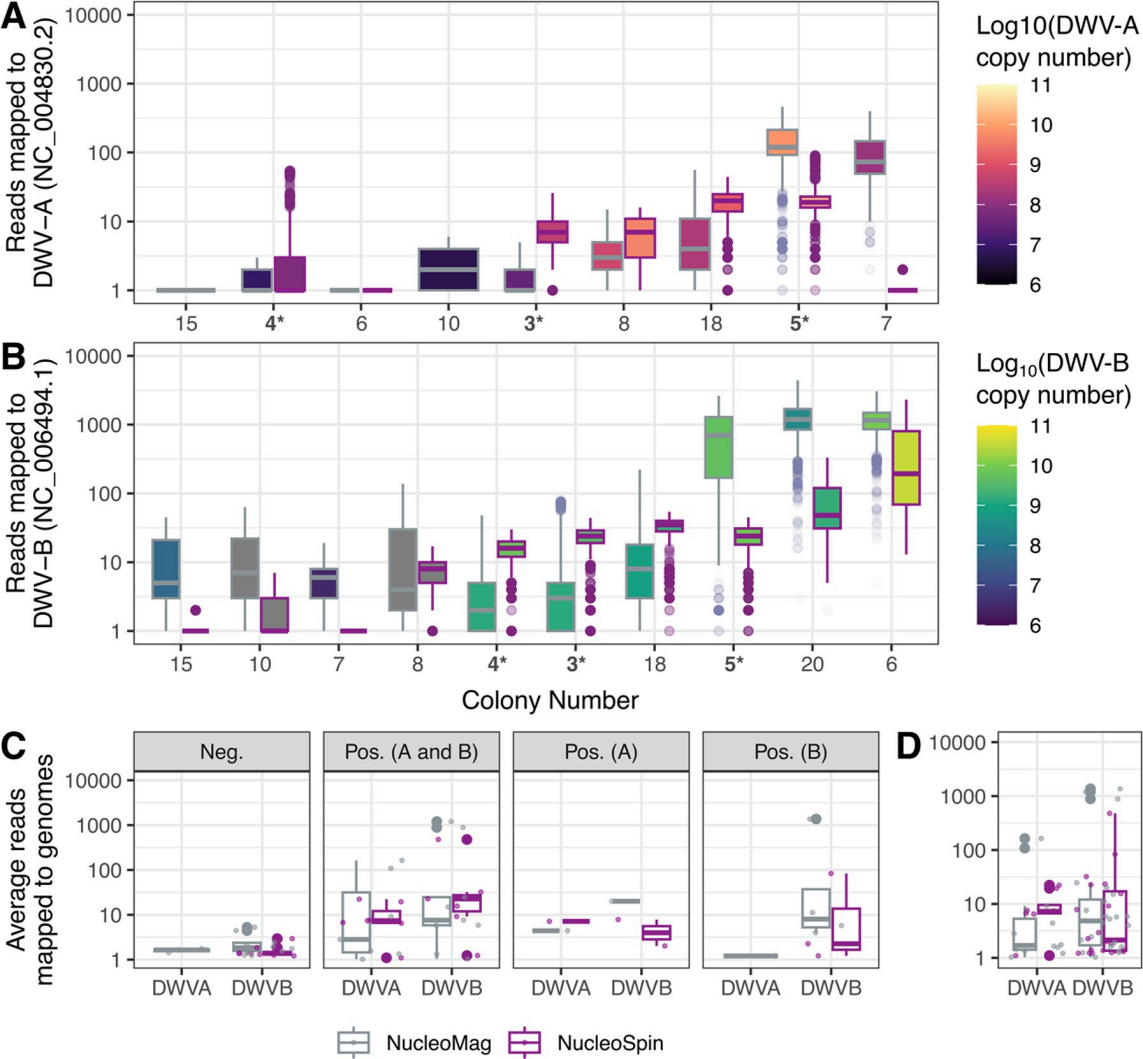
Both extraction protocols can generate high coverage of both DWV-A and DWV-B genomes that are generally in agreement with RT-qPCR data. The number of reads mapping across the reference genomes of (A) DWV-A and (B) DWV-B. Data were filtered to include only samples that mapped with >10 reads on average, removing noisy low-coverage data. The entire (unfiltered) dataset is presented as the number of reads mapped is summarized by (C) RT-qPCR detection and (D) by the total data mapped to the two genomes. In all plots genome copy numbers from the amplification of RdRp from RT-qPCR are represented as the boxplot fill color, while the line color indicates the extraction method. (Pos., Positive; Neg., Negative; *, colony sampled for DNA extraction in the year 2020).

A total of 21 contigs between 2.23 and 10.14 kbp in length, were binned initially as DWV genomes or genome fragments. After removing contigs less than 5 kbp and aligning contigs to DWV reference genomes, ten contigs were putatively classified, after BLASTn annotation, as DWV-B (min = 6.57 kbp, max = 10.14), a single DWV-A contig 7.00 kbp in length, and a two recombinant DWV contigs, one of 6.6 kbp and the other 9.7 kbp in length. Virome reads were mapped to reference DWV genomes (DWV-A, NC_004830.2; DWV-B/VDV-1, NC_006494.1). DWV-B was the most prevalent genome variant within the dataset, as demonstrated by both the abundance of DWV-B contigs assembled, proportion of sample with reads mapping to the reference genome and RT-qPCR data (Fig 3).

**Fig 4:**
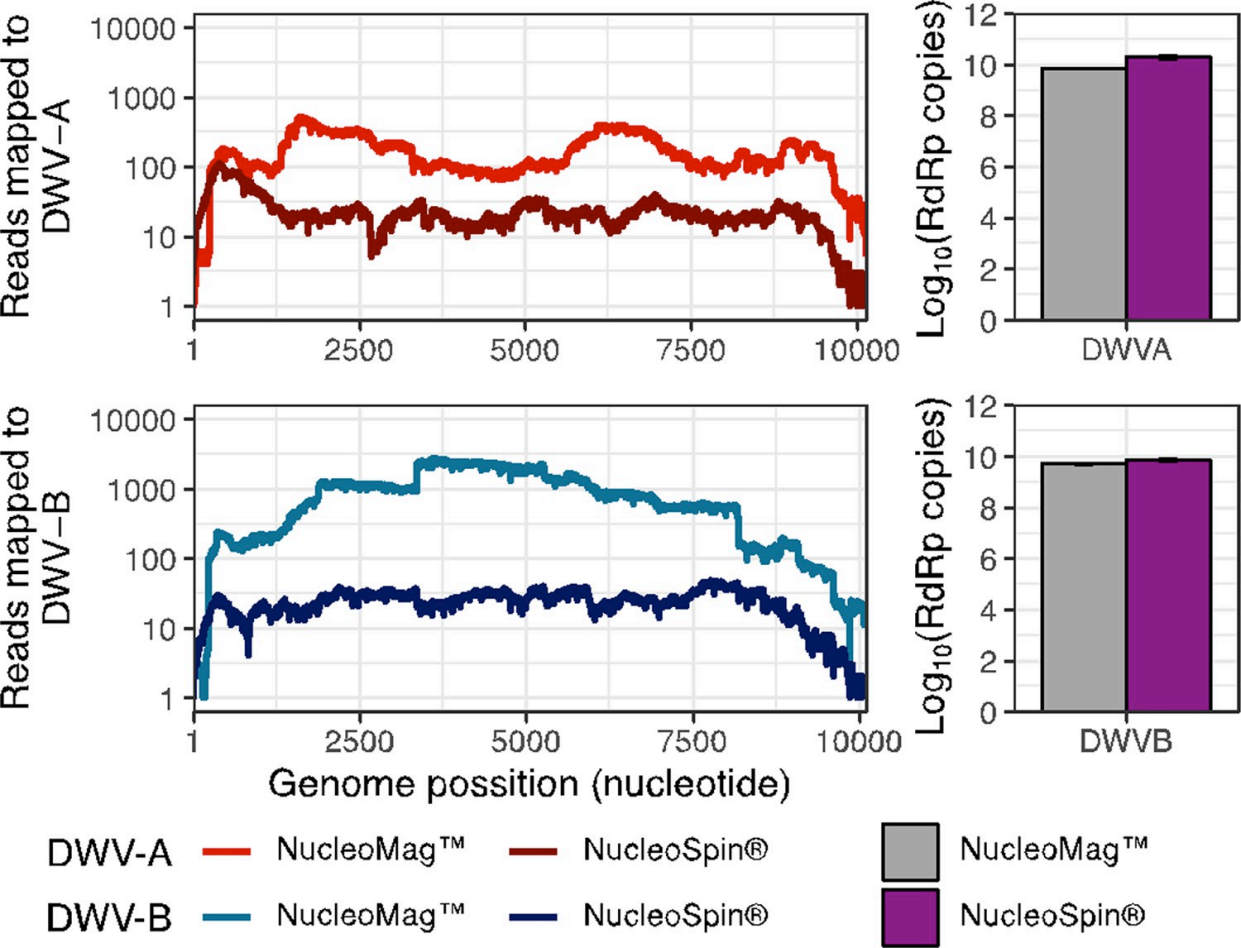
Both extraction protocols can generate high coverage of both DWV-A and DWV-B genomes that is reflected in RT-qPCR detection. Colony #5, Location: Hill, Year: 2020. Coverage histograms (left) represented as the number of reads mapped to two Deformed wing virus’ strains from NCBI, (top) DWV-A (NC_004830.2) and (bottom) DWV-B/VDV-1 (NC_006494.1). Copy number of RNA-dependent RNA polymerase for the two DWV strains as detected by RT-qPCR (right). Error bars in the barplot represent the standard deviation of the technical replicas.

Overall, we observed a similar abundance of reads mapping to both DWV-A (Fig 3A) and DWV-B (Fig 3B) from both extraction protocols (Fig 3D). A Welch t-test was performed to examine the effect of extraction protocol on the average reads per sample mapped to the reference genomes. No significant differences were found between the two extraction protocol for read mapped to DWV-A (t(26.12) = -0.711, p-value = 0.483) or DWV-B (t(27.40) = -1.351, p-value = 0.187; Fig 3D), and the means from NucleoMag™ were larger for both measures. We do observe colonies with absent mapping for one extraction method, such as colony 7 (Fig 3A). This may be explained by uneven sequencing library sizes (colony 7: NucleoSpin 1.99x105 reads and NucleoMag 6.34x105 reads). Regardless of extraction protocols, full genomes of DWV-A and B could be recovered from colonies (Fig 4). This does not always concur with the results of RT-qPCR screening, and individual inspection of the read mapping revealed likely fragmented or deteriorated genomes within the samples, as mapping did not occur around the RdRp region of the genome. Furthermore, both protocols were able to accurately identify a recombinant strain, notable due to reads mapping to regions of both DWV-A and B (Colony Number 8; Fig 3A and 3B), but detection by RT-qPCR to DWV-A (S1 Fig). In general, we observed a congruence between the RT-qPCR results and read mapping depth.

## Discussion

The NucleoMag™ extraction protocol employed in this study for isolation of viral RNA that was used for subsequent virus screening with RT-qPCR and sequencing was shown to produce as comprehensive and reproducible results as the NucleoSpin® protocol. Despite the differences in RNA yields and purity, both methods fell within an acceptable range required for successful downstream applications for accurate virus detection such as real-time virus quantification assay and cDNA library construction used for sequencing.

In this study we demonstrate that automated RNA extraction using NucleoMag™ achieves similar quality and quantity of RNA. However, with respect to sequence data, magnetic beads-based technology could exceed the quantity generated by NucleoSpin®. Spin columns technology for the extraction of total RNA from honey bees is widely used [69–71], while automated magnetic bead-based extraction protocols are not, despite benefiting from reduced manual labor (high-throughput) and improved consistency between yields.

Our data demonstrates that the sensitivity of DWV detection by both RT-qPCR and nanopore sequencing is comparable after total RNA extraction by NucleoSpin® Virus and NucleoMag™ Virus isolation kits. Both methods provided similar analytical results obtained from RT-qPCR showing that both DWV strain loads and *C*_*t*_ values from honey bee pooled samples processed by affinity purification columns and magnetic beads-based technology method correlated well, which is in agreement with previous work [37, 38]. Differences observed in the strength of amplification signal could be due to higher RNA yields and purity delivered by NucleoSpin®. In addition, sample viscosity is known to impact magnetic beads performance by impeding migration of the beads (personal communication with a manufacturer), hence impacting both the RNA yields and purity. Yet, samples extracted with NucleoSpin® generated lower sequencing data outcome, possibly due to a decreased ability of the affinity purification columns to recover small fragments of nucleic acids efficiently, as small fragments bind tightly with the silica matrix [42]. Overall, these findings provide insights into the differences RNA extraction protocol can have on the data generated from sequencing honey bee viromes.

While differences did occur in the RNA yields between the two strategies, this did not negatively impact sequencing success or achieving sufficient coverage of DWV genomes. This outcome agrees with previous work, such as a study which compared NucleoMag™ with NucleoSpin® Tissue DNA extraction kits on a range of forensic samples (e.g. human tissues), which likewise demonstrated that NucleoMag™ is suitable without compromising RNA yields or quality [72]. Elsewhere, and more relevant to the study of RNA viruses, similar results have been presented for the extraction of Enterovirus RNA, comparing automated and column extraction protocols for the purpose of RT-qPCR [73].

In general, there was a congruence between the RT-qPCR results and read mapping depth. Still, in some cases we found a mismatch between RT-qPCR and sequencing results revealing a limitation of the real-time assay to identify a recombinant DWV strain (S1 Fig, colony 8). Being a sensitive and accurate standard method for virus detection in many research fields, including honey bee research, RT-qPCR technique remains limited due to its specificity to the target genome location, while sequencing allows to target a whole genome.

Due to a small sample set we were unable to further investigate the outcome of this study in terms of viral load comparison between fall and spring samples. However, our findings confirm compatibility and accuracy of automated magnetic beads-based technology for RNA extraction and subsequent virus detection both with real-time assay and nanopore sequencing, demonstrating comparable results with manual affinity purification column method, suitable for screening up to 96 samples at a time.

## Supporting information

S1 Table 48 samples of viral RNA isolated from 24 honey bee pooled samples. Queen label represents a queen line. Viral load expressed as log10 viral RNA copies/ a pooled sample. Total sample volume was 19 ml; Sample input volume was 200ul (50ul of filtrate + 150ul of 1xPBS) and 150ul (50ul of filtrate + 100ul of 1xPBS) for magnetic beads and columns affinity extraction methods respectively.
(DOCX)

S2 Table Summary of results from multiple Welch two-sample t-tests. Significant *p*-value results (<0.05) are in bold. (M, mean; SD, standard deviation; N, number; df, degrees of freedom).
(DOCX)

S1 Fig Both extraction protocols are capable of identifying chimeric DWV genomes. Colony # 8, Location: Hill, Year: 2021. Coverage histograms (left) represented as the number of reads mapped to two Deformed wing virus’ strains from NCBI, (top) DWV-A (NC_004830.2) and (bottom) DWV-B/VDV-1 (NC_006494.1). Copy number of RNA-dependent RNA polymerase for the two DWV strains as detected by RT-qPCR (right).
(DOCX)

## References

[1] Klein AM, Vaissière BE, Cane JH, Steffan-Dewenter I, Cunningham SA, Kremen C, et al. Importance of pollinators in changing landscapes for world crops. Proc Biol Sci. 2007 Feb 7;274(1608):303–13. doi: 10.1098/rspb.2006.3721 17164193 PMC1702377

[2] Hung KLJ, Kingston JM, Albrecht M, Holway DA, Kohn JR. The worldwide importance of honey bees as pollinators in natural habitats. Proc Biol Sci. 2018 Jan 10;285(1870):20172140. doi: 10.1098/rspb.2017.2140 29321298 PMC5784195

[3] Calderone NW. Insect Pollinated Crops, Insect Pollinators and US Agriculture: Trend Analysis of Aggregate Data for the Period 1992–2009. PLOS ONE. 2012 May 22;7(5):e37235. doi: 10.1371/journal.pone.0037235 22629374 PMC3358326

[4] Gallai N, Salles JM, Settele J, Vaissière BE. Economic valuation of the vulnerability of world agriculture confronted with pollinator decline. Ecological Economics. 2009 Jan 15;68(3):810–21.

[5] Gray A, Adjlane N, Arab A, Ballis A, Brusbardis V, Bugeja Douglas A, et al. Honey bee colony loss rates in 37 countries using the COLOSS survey for winter 2019–2020: the combined effects of operation size, migration and queen replacement. Journal of Apicultural Research. 2023 Mar 15;62(2):204–10.

[6] Paudel Y, Mackereth R, Hanley R, Qin W. Honey Bees (Apis mellifera L.) and Pollination Issues: Current status, impacts and potential drivers of decline. Journal of Agricultural Science. 2015 May 15;7(6):p93.

[7] Potts SG, Roberts SPM, Dean R, Marris G, Brown MA, Jones R, et al. Declines of managed honey bees and beekeepers in Europe. Journal of Apicultural Research. 2010 Jan;49(1):15–22.

[8] Spivak M, Mader E, Vaughan M, Euliss NH. The plight of the bees. Environ Sci Technol. 2011 Jan 1;45(1):34–8. doi: 10.1021/es101468w 20839858

[9] Goulson D, Nicholls E, Botías C, Rotheray EL. Bee declines driven by combined stress from parasites, pesticides, and lack of flowers. Science. 2015 Mar 27;347(6229):1255957. doi: 10.1126/science.1255957 25721506

[10] Sánchez-Bayo F, Wyckhuys KAG. Worldwide decline of the entomofauna: A review of its drivers. Biological Conservation. 2019 Apr;232:8–27.

[11] Martin SJ, Highfield AC, Brettell L, Villalobos EM, Budge GE, Powell M, et al. Global honey bee viral landscape altered by a parasitic mite. Science. 2012 Jun 8;336(6086):1304–6. doi: 10.1126/science.1220941 22679096

[12] Traynor KS, Mondet F, de Miranda JR, Techer M, Kowallik V, Oddie MAY, et al. Varroa destructor: A Complex Parasite, Crippling Honey Bees Worldwide. Trends in Parasitology. 2020 Jul 1;36(7):592–606. doi: 10.1016/j.pt.2020.04.004 32456963

[13] Beaurepaire A, Piot N, Doublet V, Antunez K, Campbell E, Chantawannakul P, et al. Diversity and Global Distribution of Viruses of the Western Honey Bee, Apis mellifera. Insects. 2020 Apr;11(4):239. doi: 10.3390/insects11040239 32290327 PMC7240362

[14] Gisder S, Genersch E. Special Issue: Honey Bee Viruses. Viruses. 2015 Oct;7(10):5603–8. doi: 10.3390/v7102885 26702462 PMC4632393

[15] McMenamin AJ, Flenniken ML. Recently identified bee viruses and their impact on bee pollinators. Curr Opin Insect Sci. 2018 Apr;26:120–9. doi: 10.1016/j.cois.2018.02.009 29764651

[16] Yañez O, Piot N, Dalmon A, De Miranda JR, Chantawannakul P, Panziera D, et al. Bee Viruses: Routes of Infection in Hymenoptera. Front Microbiol. 2020 May 28;11:943. doi: 10.3389/fmicb.2020.00943 32547504 PMC7270585

[17] Highfield AC, El Nagar A, Mackinder LCM, Noël LMLJ, Hall MJ, Martin SJ, et al. Deformed Wing Virus Implicated in Overwintering Honeybee Colony Losses. Applied and Environmental Microbiology. 2009 Nov;75(22). doi: 10.1128/AEM.02227-09 19783750 PMC2786540

[18] Martin SJ, Brettell LE. Deformed Wing Virus in Honeybees and Other Insects. Annu Rev Virol. 2019 Sep 29;6(1):49–69. doi: 10.1146/annurev-virology-092818-015700 31185188

[19] Levin S, Sela N, Erez T, Nestel D, Pettis J, Neumann P, et al. New Viruses from the Ectoparasite Mite Varroa destructor Infesting Apis mellifera and Apis cerana. Viruses. 2019 Jan 24;11(2):94. doi: 10.3390/v11020094 30678330 PMC6409542

[20] Paxton RJ, Schäfer MO, Nazzi F, Zanni V, Annoscia D, Marroni F, et al. Epidemiology of a major honey bee pathogen, deformed wing virus: potential worldwide replacement of genotype A by genotype B. Int J Parasitol Parasites Wildl. 2022 Aug;18:157–71. doi: 10.1016/j.ijppaw.2022.04.013 35592272 PMC9112108

[21] Kevill JL, de Souza FS, Sharples C, Oliver R, Schroeder DC, Martin SJ. DWV-A Lethal to Honey Bees (Apis mellifera): A Colony Level Survey of DWV Variants (A, B, and C) in England, Wales, and 32 States across the US. Viruses. 2019 May;11(5). 10.3390/v11050426 PMC6563202 31075870

[22] Forfert N, Natsopoulou ME, Frey E, Rosenkranz P, Paxton RJ, Moritz RFA. Parasites and Pathogens of the Honeybee (Apis mellifera) and Their Influence on Inter-Colonial Transmission. PLOS ONE. 2015 Oct;10(10). doi: 10.1371/journal.pone.0140337 26451849 PMC4599887

[23] Alger SA, Burnham PA, Lamas ZS, Brody AK, Richardson LL. Home sick: impacts of migratory beekeeping on honey bee (*Apis mellifera*) pests, pathogens, and colony size. PeerJ. 2018 Nov;6. 10.7717/peerj.5812 PMC6216951 30405967

[24] Dynes TL, Berry JA, Delaplane KS, Brosi BJ, de Roode JC. Reduced density and visually complex apiaries reduce parasite load and promote honey production and overwintering survival in honey bees. PLOS ONE. 2019 May;14(5). doi: 10.1371/journal.pone.0216286 31120911 PMC6532956

[25] De Miranda JR, Bailey L, Ball BV, Blanchard P, Budge GE, Chejanovsky N, et al. Standard methods for virus research in *Apis mellifera*. Journal of Apicultural Research. 2013 Jan;52(4):1–56.

[26] Li M, Sun L, Ma Y, Fei D, Ma M. Development of a sandwich ELISA for the detection of Chinese sacbrood virus infection. Arch Virol. 2020 Jul;165(7):1551–6. doi: 10.1007/s00705-020-04634-2 32356186

[27] Glover RH, Adams IP, Budge G, Wilkins S, Boonham N. Detection of honey bee (Apis mellifera) viruses with an oligonucleotide microarray. J Invertebr Pathol. 2011 Jul;107(3):216–9. doi: 10.1016/j.jip.2011.03.004 21419132

[28] Goblirsch MJ, Spivak MS, Kurtti TJ. A cell line resource derived from honey bee (Apis mellifera) embryonic tissues. PLoS One. 2013;8(7):e69831. doi: 10.1371/journal.pone.0069831 23894551 PMC3720946

[29] Guo Y, Goodman CL, Stanley DW, Bonning BC. Cell Lines for Honey Bee Virus Research. Viruses. 2020 Feb 20;12(2):236. 32093360 10.3390/v12020236 PMC7077248

[30] Tentcheva D, Gauthier L, Bagny L, Fievet J, Dainat B, Cousserans F, et al. Comparative analysis of deformed wing virus (DWV) RNA in *Apis mellifera* and *Varroa destructor*. Apidologie. 2006 Jan;37(1):41–50.

[31] Levin S, Galbraith D, Sela N, Erez T, Grozinger CM, Chejanovsky N. Presence of Apis Rhabdovirus-1 in Populations of Pollinators and Their Parasites from Two Continents. Front Microbiol. 2017 Dec 12;8:2482. doi: 10.3389/fmicb.2017.02482 29312191 PMC5732965

[32] Liu S, Vijayendran D, Bonning BC. Next Generation Sequencing Technologies for Insect Virus Discovery. Viruses. 2011 Oct 10;3(10):1849–69. doi: 10.3390/v3101849 22069519 PMC3205385

[33] Yu L, Adamson P, Lay Yap P, Tung T, Makar S, Turra M, et al. From Biowaste to Lab-Bench: Low-Cost Magnetic Iron Oxide Nanoparticles for RNA Extraction and SARS-CoV-2 Diagnostics. Biosensors. 2023 Jan;13(2):196–196. doi: 10.3390/bios13020196 36831962 PMC9953475

[34] Palmer EJ, Maestre JP, Jarma D, Lu A, Willmann E, Kinney KA, et al. Development of a reproducible method for monitoring SARS-CoV-2 in wastewater. Sci Total Environ. 2021 Dec 10;799:149405. doi: 10.1016/j.scitotenv.2021.149405 34365266 PMC8328530

[35] He H, Li R, Chen Y, Pan P, Tong W, Dong X, et al. Integrated DNA and RNA extraction using magnetic beads from viral pathogens causing acute respiratory infections. Scientific Reports. 2017 Mar;7(1):45199–45199. doi: 10.1038/srep45199 28332631 PMC5362898

[36] Scarabotto A, Balestro S, Gagliardi S, Trotti R. Comparison of Two RNA Extraction Methods for the Molecular Detection of SARS-CoV-2 from Nasopharyngeal Swab Samples. Diagnostics. 2022 Jun;12(7):1561–1561. doi: 10.3390/diagnostics12071561 35885467 PMC9317615

[37] Hongjaisee S, Jabjainai Y, Sakset S, Preechasuth K, Ngo-Giang-Huong N, Khamduang W. Comparison of Simple RNA Extraction Methods for Molecular Diagnosis of Hepatitis C Virus in Plasma. Diagnostics. 2022 Jun;12(7):1599–1599. doi: 10.3390/diagnostics12071599 35885505 PMC9322174

[38] Dang T, Bodaghi S, Osman F, Wang J, Rucker T, Tan SH, et al. A comparative analysis of RNA isolation methods optimized for high-throughput detection of viral pathogens in California’s regulatory and disease management program for citrus propagative materials. Frontiers in Agronomy. 2022 Aug;4.

[39] Brettell LE, Schroeder DC, Martin SJ. RNAseq of Deformed Wing Virus and Other Honey Bee-Associated Viruses in Eight Insect Taxa with or without Varroa Infestation. Viruses. 2020 Oct 29;12(11):1229. doi: 10.3390/v12111229 33138298 PMC7692275

[40] Levin S, Sela N, Chejanovsky N. Two novel viruses associated with the Apis mellifera pathogenic mite Varroa destructor. Sci Rep. 2016 Nov 24;6:37710. doi: 10.1038/srep37710 27883042 PMC5121581

[41] Galbraith DA, Fuller ZL, Ray AM, Brockmann A, Frazier M, Gikungu MW, et al. Investigating the viral ecology of global bee communities with high-throughput metagenomics. Sci Rep. 2018 Jun 11;8(1):8879. doi: 10.1038/s41598-018-27164-z 29891995 PMC5995813

[42] Schmitz TC, Dede Eren A, Spierings J, de Boer J, Ito K, Foolen J. Solid-phase silica-based extraction leads to underestimation of residual DNA in decellularized tissues. Xenotransplantation. 2021 Jan;28(1):e12643. doi: 10.1111/xen.12643 32935355 PMC9286341

[43] Evans JD, Schwarz RS, Chen YP, Budge G, Cornman RS, De la Rua P, et al. Standard methods for molecular research in Apis mellifera. Journal of Apicultural Research. 2013 Jan;52(4).

[44] Olgun T, Everhart SE, Anderson T, Wu-Smart J. Comparative analysis of viruses in four bee species collected from agricultural, urban, and natural landscapes. PLOS ONE. 2020 Jun;15(6). doi: 10.1371/journal.pone.0234431 32530936 PMC7292363

[45] Traynor KS, Rennich K, Forsgren E, Rose R, Pettis J, Kunkel G, et al. Multiyear survey targeting disease incidence in US honey bees. Apidologie. 2016 May;47(3).

[46] Dalmon A, Diévart V, Thomasson M, Fouque R, Vaissière BE, Guilbaud L, et al. Possible Spillover of Pathogens between Bee Communities Foraging on the Same Floral Resource. Insects. 2021 Jan;12(2). doi: 10.3390/insects12020122 33573084 PMC7911050

[47] Piot N, Schweiger O, Meeus I, Yañez O, Straub L, Villamar-Bouza L, et al. Honey bees and climate explain viral prevalence in wild bee communities on a continental scale. Scientific Reports. 2022 Feb;12(1). doi: 10.1038/s41598-022-05603-2 35115568 PMC8814194

[48] Kandel M, Paxton RJ, Al Naggar Y. Nationwide Screening for Bee Viruses in Apis mellifera Colonies in Egypt. Insects. 2023 Feb;14(2). doi: 10.3390/insects14020172 36835740 PMC9964814

[49] Locke B, Forsgren E, Fries I, de Miranda JR. Acaricide Treatment Affects Viral Dynamics in Varroa destructor-Infested Honey Bee Colonies via both Host Physiology and Mite Control. Applied and Environmental Microbiology. 2012 Jan;78(1). doi: 10.1128/AEM.06094-11 22020517 PMC3255632

[50] Truong AT, Yoo MS, Seo SK, Hwang TJ, Yoon SS, Cho YS. Prevalence of honey bee pathogens and parasites in South Korea: A five-year surveillance study from 2017 to 2021. Heliyon. 2023 Feb 4;9(2):e13494. doi: 10.1016/j.heliyon.2023.e13494 36816323 PMC9929316

[51] Dhibika M, Madhusudhan NS, Malini A, Natarajan M. Comparison of Manual and Automated Nucleic Acid (RNA) Extraction Methods for the Detection of SARS-CoV-2 by qRT-PCR. Cureus. 2023 Mar;15(3):e36773. doi: 10.7759/cureus.36773 37123735 PMC10133768

[52] Rogers SO, editor. Molecular analyses. First edition. Boca Raton: CRC Press, Taylor & Francis Group; 2022. 361 p. (Molecular genomics and proteomics).

[53] Ward L, Waite R, Boonham N, Fisher T, Pescod K, Thompson H, et al. First detection of Kashmir bee virus in the UK using real-time PCR. Apidologie. 2007 Mar;38(2):181–90.

[54] Amiri E, Kryger P, Meixner MD, Strand MK, Tarpy DR, Rueppell O. Quantitative patterns of vertical transmission of deformed wing virus in honey bees. Nieh JC, editor. PLoS ONE. 2018 Mar 29;13(3):e0195283. doi: 10.1371/journal.pone.0195283 29596509 PMC5875871

[55] Francis RM, Nielsen SL, Kryger P. Varroa-Virus Interaction in Collapsing Honey Bee Colonies. Martin SJ, editor. PLoS ONE. 2013 Mar 19;8(3):e57540. doi: 10.1371/journal.pone.0057540 23526946 PMC3602523

[56] Penn HJ, Simone-Finstrom MD, De Guzman LI, Tokarz PG, Dickens R. Colony-Level Viral Load Influences Collective Foraging in Honey Bees. Front Insect Sci. 2022 May 17;2:894482. 38468777 10.3389/finsc.2022.894482 PMC10926460

[57] Kevill JL, Highfield A, Mordecai GJ, Martin SJ, Schroeder DC. ABC Assay: Method Development and Application to Quantify the Role of Three DWV Master Variants in Overwinter Colony Losses of European Honey Bees. Viruses. 2017 Oct 27;9(11):314. doi: 10.3390/v9110314 29077069 PMC5707521

[58] Wick R, Volkening J, Loman N. Porechop: adapter trimmer for Oxford Nanopore reads. https://github.com/rrwick/Porechop/ (August 2022, date last accessed). 2017.

[59] Koren S, Walenz BP, Berlin K, Miller JR, Bergman NH, Phillippy AM. Canu: scalable and accurate long-read assembly via adaptive k-mer weighting and repeat separation. Genome Res. 2017 May;27(5):722–36. doi: 10.1101/gr.215087.116 28298431 PMC5411767

[60] Eren AM, Esen ÖC, Quince C, Vineis JH, Morrison HG, Sogin ML, et al. Anvi’o: an advanced analysis and visualization platform for ‘omics data. PeerJ. 2015;3:e1319. doi: 10.7717/peerj.1319 26500826 PMC4614810

[61] Eren AM, Kiefl E, Shaiber A, Veseli I, Miller SE, Schechter MS, et al. Community-led, integrated, reproducible multi-omics with anvi’o. Nat Microbiol. 2021 Jan;6(1):3–6. doi: 10.1038/s41564-020-00834-3 33349678 PMC8116326

[62] Hyatt D, Chen GL, Locascio PF, Land ML, Larimer FW, Hauser LJ. Prodigal: prokaryotic gene recognition and translation initiation site identification. BMC Bioinformatics. 2010 Mar 8;11:119. doi: 10.1186/1471-2105-11-119 20211023 PMC2848648

[63] Li H. Minimap2: pairwise alignment for nucleotide sequences. Bioinformatics. 2018 Sep 15;34(18):3094–100. doi: 10.1093/bioinformatics/bty191 29750242 PMC6137996

[64] Bedre R. Bioinformatics data analysis and visualization toolkit. reneshbedre/bioinfokit: Zenodo; 2022.

[65] Aramaki T, Blanc-Mathieu R, Endo H, Ohkubo K, Kanehisa M, Goto S, et al. KofamKOALA: KEGG Ortholog assignment based on profile HMM and adaptive score threshold. Bioinformatics. 2020 Apr 1;36(7):2251–2. doi: 10.1093/bioinformatics/btz859 31742321 PMC7141845

[66] Wood DE, Salzberg SL. Kraken: ultrafast metagenomic sequence classification using exact alignments. Genome Biol. 2014 Mar 3;15(3):R46. doi: 10.1186/gb-2014-15-3-r46 24580807 PMC4053813

[67] Wood DE, Lu J, Langmead B. Improved metagenomic analysis with Kraken 2. Genome Biology. 2019 Nov 28;20(1):257. doi: 10.1186/s13059-019-1891-0 31779668 PMC6883579

[68] Shen W, Le S, Li Y, Hu F. SeqKit: A Cross-Platform and Ultrafast Toolkit for FASTA/Q File Manipulation. PLoS One. 2016 Oct 5;11(10):e0163962. doi: 10.1371/journal.pone.0163962 27706213 PMC5051824

[69] de Souza FS, Kevill JL, Correia-Oliveira ME, de Carvalho CAL, Martin SJ. Occurrence of deformed wing virus variants in the stingless bee Melipona subnitida and honey bee Apis mellifera populations in Brazil. J Gen Virol. 2019 Feb;100(2):289–94. doi: 10.1099/jgv.0.001206 30628883

[70] Gusachenko ON, Woodford L, Balbirnie-Cumming K, Ryabov EV, Evans DJ. Evidence for and against deformed wing virus spillover from honey bees to bumble bees: a reverse genetic analysis. Sci Rep. 2020 Oct 8;10(1):16847. doi: 10.1038/s41598-020-73809-3 33033296 PMC7546617

[71] Posada-Florez F, Childers AK, Heerman MC, Egekwu NI, Cook SC, Chen Y, et al. Deformed wing virus type A, a major honey bee pathogen, is vectored by the mite Varroa destructor in a non-propagative manner. Sci Rep. 2019 Aug 27;9(1):12445. doi: 10.1038/s41598-019-47447-3 31455863 PMC6712216

[72] D’Onofrio A, Crawford JM, Stewart EJ, Witt K, Gavrish E, Epstein S, et al. Siderophores from neighboring organisms promote the growth of uncultured bacteria. Chem Biol. 2010 Mar 26;17(3):254–64. doi: 10.1016/j.chembiol.2010.02.010 20338517 PMC2895992

[73] Knepp JH, Geahr MA, Forman MS, Valsamakis A. Comparison of automated and manual nucleic acid extraction methods for detection of enterovirus RNA. J Clin Microbiol. 2003 Aug;41(8):3532–6. doi: 10.1128/JCM.41.8.3532-3536.2003 12904351 PMC179781

