# Supplementary material for: A semi-automated and high-throughput approach for the detection of honey bee viruses in bee samples
Source: PLoS One. 2024 Mar 14;19(3):e0297623. doi: 10.1371/journal.pone.0297623 (PMC10939240; doi:10.1371/journal.pone.0297623)
Supplement: S1 Table — Queen label represents a queen line. Viral load expressed as log10 viral RNA copies/ a pooled sample. Total sample volume was 19 ml; Sample input volume was 200ul (50ul of filtrate + 150ul of 1xPBS) and 150ul (50ul of filtrate + 100ul of 1xPBS) for magnetic beads and columns affinity extraction methods respectively. (DOCX) [file pone.0297623.s001.docx]

**S1 Table. 48 samples of viral RNA isolated from 24 honey bee pooled samples.** Queen label represents a queen line. Viral load expressed as log10 viral RNA copies/ a pooled sample. Total sample volume was 19 ml; Sample input volume was 200ul (50ul of filtrate + 150ul of 1xPBS) and 150ul (50ul of filtrate + 100ul of 1xPBS) for magnetic beads and columns affinity extraction methods respectively.
