# Supplementary material for: A semi-automated and high-throughput approach for the detection of honey bee viruses in bee samples
Source: PLoS One. 2024 Mar 14;19(3):e0297623. doi: 10.1371/journal.pone.0297623 (PMC10939240; doi:10.1371/journal.pone.0297623)
Supplement: S2 Table — Significant p-value results (<0.05) are in bold. (M, mean; SD, standard deviation; N, number; df, degrees of freedom). (DOCX) [file pone.0297623.s002.docx]

**S2 Table. Summary of results from multiple Welch two-sample t-tests.**Significant*p*-value results (<0.05) are in bold. (M, mean; SD, standard deviation; N, number; df, degrees of freedom).

|  | **NucleoMag™** | **NucleoSpin®** | **T-value (df)** | **p-value** |
| --- | --- | --- | --- | --- |
| **RNA yield (ng/ul)** | M=55.7, SD=22.9, N=24 | M=122, SD=69.4, N=24 | -4.4263 (45) | **0.000133** |
| **260/280 ratio** | M=2.09, SD=0.043, N=24 | M=2.12, SD=0.0383, N=24 | -2.329 (45) | **0.02592** |
| **260/230 ratio** | M=1.77, SD=0.226, N=24 | M=2.05, SD=0.408, N=24 | -2.9663 (45) | **0.005334** |
| **Number of reads** | M=113719, SD=95302, N=24 | M=41098, SD=41995, N=24 | 3.4161 (45) | **0.001338** |
| **Length of reads** | M=388, SD=221, N=897139 | M=398, SD=220,  N=83851 | -12.582 (100275) | **<2.2x10^-16^** |
| **Average read quality** | M=12.8 , SD=2.05, N=897139 | M=12.3, SD=2.00, N=83851 | 69.535  (100998) | **<2.2x10^-16^** |
| **Number of contigs** | M=80.2, SD=65.4, N=18 | M=24.2, SD=15.4, N=24 | 3.5612 (18.427) | **0.002166** |
| **Length of contigs** | M=5578, SD=5144, N=425 | M=5585, SD=5066, N=364 | -0.01815 (771.86) | 0.9855 |
| **Maping to DWV-A (NC_004830.2)** | M=14.6, SD=42.5, N=24 | M=8.18, SD=7.72, N=11 | -0.711 (26.12) | 0.483 |
| **Maping to DWV-B (NC_006494.1)** | M=164, SD=433, N=24 | M=38.7, SD=129, N=22 | -1.351 (27.40) | 0.187 |
