# Supplementary material for: A semi-automated and high-throughput approach for the detection of honey bee viruses in bee samples
Source: PLoS One. 2024 Mar 14;19(3):e0297623. doi: 10.1371/journal.pone.0297623 (PMC10939240; doi:10.1371/journal.pone.0297623)
Supplement: S1 Fig — Colony # 8, Location: Hill, Year: 2021. Coverage histograms (left) represented as the number of reads mapped to two Deformed wing virus’ strains from NCBI, (top) DWV-A (NC_004830.2) and (bottom) DWV-B/VDV-1 (NC_006494.1). Copy number of RNA-dependent RNA polymerase for the two DWV strains as detected by RT-qPCR (right). (DOCX) [file pone.0297623.s003.docx]

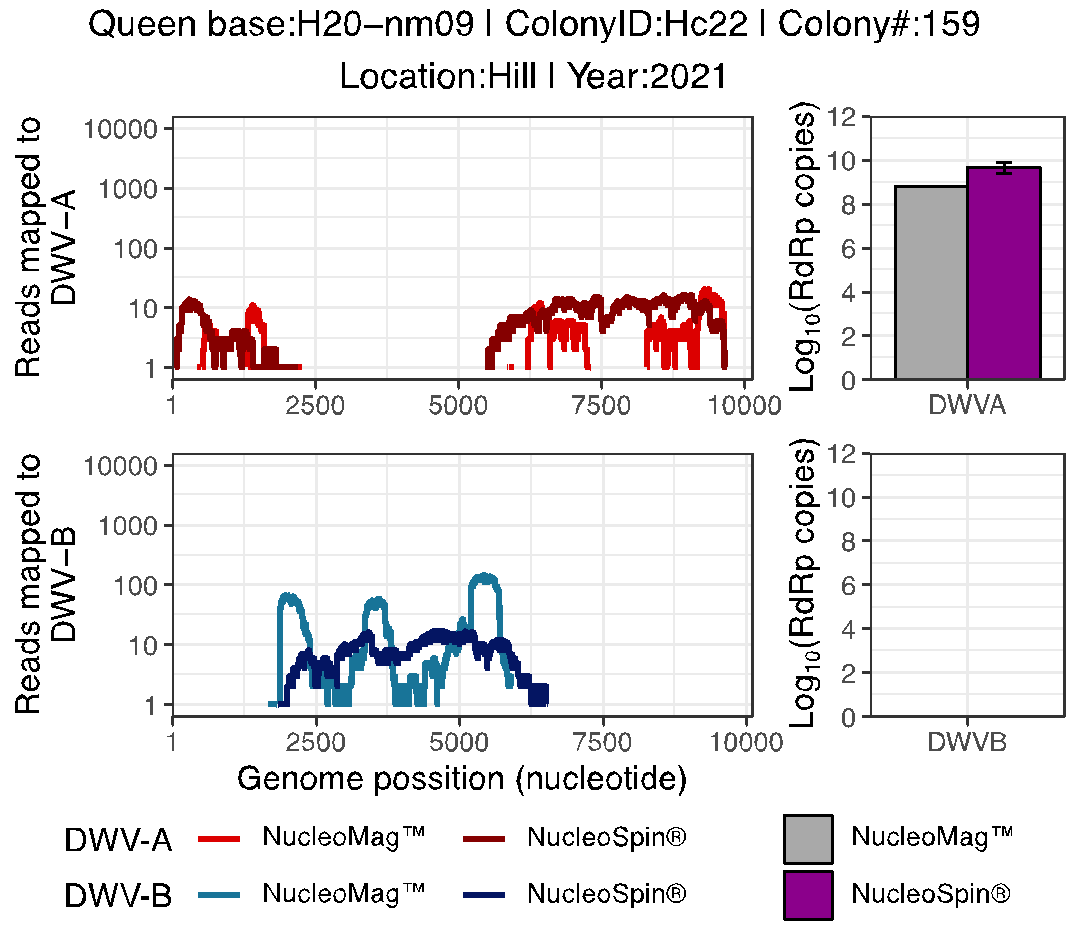


**S1 Fig. Both extraction protocols are capable of identifying chimeric DWV genomes.**

Colony # 8, Location: Hill, Year: 2021. Coverage histograms (left) represented as the number of reads mapped to two Deformed wing virus' strains from NCBI, (top) DWV-A (NC_004830.2) and (bottom) DWV-B/VDV-1 (NC_006494.1). Copy number of  RNA-dependent RNA polymerase for the two DWV strains as detected by RT-qPCR (right).
